# Electronic teaching files and continuing professional development in radiology

**DOI:** 10.2349/biij.2.2.e5

**Published:** 2006-04-01

**Authors:** CCT Lim, GL Yang

**Affiliations:** 1 Department of Neuroradiology, National Neuroscience Institute, Singapore; 2 Biomedical Imaging Laboratory, Agency for Science Technology and Research, Singapore

**Keywords:** Radiology education, radiology information systems, medical imaging resource centre

## Abstract

Education in diagnostic radiology employs medical images extensively, and case-based teaching files of actual patients are useful to illustrate pertinent teaching points. In the era of digital radiology, there is great potential to use the ready source of patient material from Picture Archive and Communication Systems (PACS) and initiatives such as Medical Imaging Resource Center (MIRC) on the World Wide Web for teaching and for Continuing Professional Development (CPD). As mandatory CPD becomes the reality in medical practice, computerised solutions that support the creation of electronic teaching files in the midst of busy clinical workflow would be very valuable. This paper will explore the features of image-based CPD, the various ways in which medical images can be used for self directed learning and the challenges that face the radiology profession.

## INTRODUCTION

Continuing Professional Development (CPD) is becoming increasingly important for medical professionals. In the 1990’s, Continuing Medical Education (CME) initiatives were in their infancy and served to enable practicing physicians to keep up with current advances and state-of-the-art practice of medicine. With growing public attention and awareness, there has been increasing pressure to maintain standards of patient care and for regulation and oversight of the medical profession [1-6]. Physician performance and outcome measurements are of interest to the profession and increasing concern to the public as well as to external organisations such as the Joint Commission on Accreditation of Healthcare Organisations. In many countries, CME programmes have developed and changed to become professional revalidation and maintenance of certification programmes. In the United States, mandatory maintenance of re-certification (including an examination), has resulted in increased demand for programs and infrastructure for lifelong learning and self-assessment [6].

In order to deliver CPD effectively in the context of busy medical practice, on-demand remote or distance learning initiative would be desirable, particularly exploiting the advantages of computerisation and the World Wide Web. Like other subspecialties, diagnostic radiology also faces demands from CPD. However, the unique features and challenges of education in diagnostic images make this at the same time a daunting task and an unparalleled opportunity.

## RADIOLOGY EDUCATION AND TEACHING FILES

Medical education in diagnostic radiology is heavily image-intensive. In order to distinguish the subtle differences between diseases, diagnostic radiologists need to learn and master a large template of abnormal findings, normal features, and normal findings that mimic disease. Visual “Aunt Minnies” (like your own aunt, it may be easier to recognize her than to describe her), with unique features that allow immediate diagnosis of a certain disease are another highlight of radiology learning [7].

The ability to identify and appreciate the subtle nuances in the difference between diseases that often have similar imaging findings but vastly different treatments and outcomes, is honed by continued exposure to a large number of representative images. This has traditionally been accomplished during radiology specialty training by rotating through various imaging departments. It would be desirable to distil this accumulated experience and training into an organised body of images with educational value, which is in the form of radiological teaching files. Such case based teaching files can be derived from images from real patients that illustrate a particular teaching point, diagnostic pitfall or unexpected outcome. Most radiology departments in teaching hospitals already maintain a collection of teaching files that comprise, at the most basic level, a number of medical images, and pertinent information about the clinical details, diagnosis, outcome and perhaps a short discourse on the nature of the disease and its associated imaging findings. This may be supplemented by appropriate references and suggested reading, differential diagnosis and pearls of wisdom. In the past, these teaching files would be stored as hard copy film and paper and filed according to a classification system such as the American College of Radiologists index of radiological diagnosis [8].

## ELECTRONIC TEACHING FILES AND PACS

Hardcopy films have several drawbacks in that they are prone to physical degradation, can be used by only one person or one group at a time, occupy physical space, and are apt to be misplaced. With the advent of computers and the digital age, the acquisition of natively digital radiological images (such as computed tomography and magnetic resonance images that were created by computer processing as opposed to analogue film-screen exposure) has become an irresistible trend. Such data can be manipulated, archived, and transmitted with much greater ease and convenience in the digital realm, and Picture Archive and Communication Systems (PACS) are impacting radiology workflow, cost and productivity. Although the advent of PACS will result in the decline (and demise) of the physical film library, to date there have been no commercial PACS vendors that have developed a full-functional teaching file solution. Several workarounds have been proposed, and resources and authoring tools are available for making “teaching folders” or exporting relevant images [9-15].

## WORLD WIDE WEB EDUCATIONAL RESOURCES

**Figure 1:**
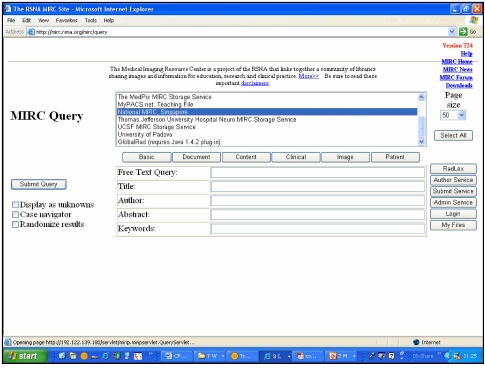
Medical Imaging Resource Center (MIRC) web portal (http://mirc.rsna.org/mirc/query) of the Radiological Society of North America. This query service links multiple participating MIRC sites, allowing users to search for electronic teaching files.

There are many online repositories of electronic radiology educational material available on the World Wide Web [16-19], with several sites offering teaching files as “Case of the Day”. More sophisticated initiatives on the World Wide Web include the search engines such as Medical Imaging Resource Center (MIRC) of the Radiological Society of North America (RSNA) [20-22]. This initiative goes beyond merely offering a collection of radiological images, and establishes a set of electronic schema (based on Extensible Markup Language, or XML) to define electronic teaching files for search engines. Any MIRC site can function as a query service or storage service or both. In the query service, a MIRC web portal will be able to search all the linked MIRC storage websites for medical images (for instance based on free text search). The storage service will then respond and return links to matching electronic teaching files. The MIRC project envisages a worldwide community of radiologists that will share medical images (conforming to the schema), enabling seamless communication of images for education and research purposes. A number of institutions and internet-based teaching files are currently linked to MIRC (Figure 1).

**Figure 2:**
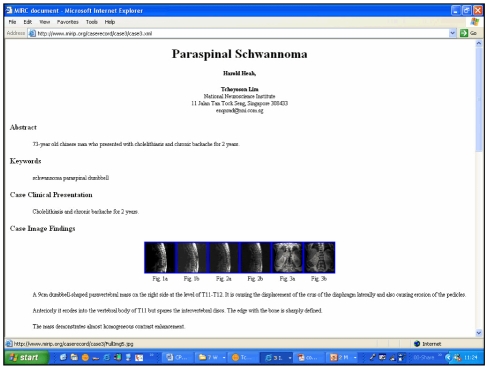
Example of a MIRC electronic teaching file created on MIRIP using images from PACS (see text).

In order to take advantage of the rich source of images afforded by PACS [23] and the potential of an internet search engine of MIRC, our group has developed Medical Imaging Resource Interface with PACS (MIRIP) [24]. This solution is essentially a computer server running a database programme, an image server and a web server that enables users to generate an electronic teaching file from an existing patient’s images in PACS. To conform to relevant privacy laws [25] and patient confidentiality, all images are anonymous. Teaching files generated by MIRIP are in conformance to the MIRC schema and may be viewed on World Wide Web and can participate in the RSNA search engine (Figure 2).

**Figure 3:**
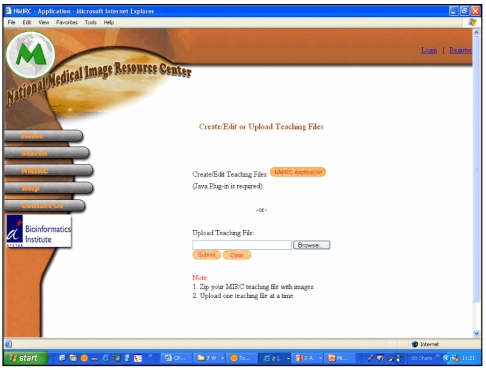
The Singapore National MIRC website (http://www.mirip.org/nmirc.jsp) with MIRC-compliant query and storage services, which also allows users to upload or create electronic teachingfiles online.

In addition to the enterprise-wide MIRIP designed for large teaching hospitals, our group has also built a range of teaching file authoring tools, including a “lite” version for personal use as a radiologist’s case library [26], as well as an online submission system for radiologists who do not have either the MIRC enterprise or personal versions, but who would like to participate in creating electronic teaching files on the World Wide Web [27]. In this way, we have created a Singapore National MIRC initiative which is linked to RSNA MIRC (Figure 3).

## ELECTRONIC TEACHING FILES FOR CPD

**Figure 4:**
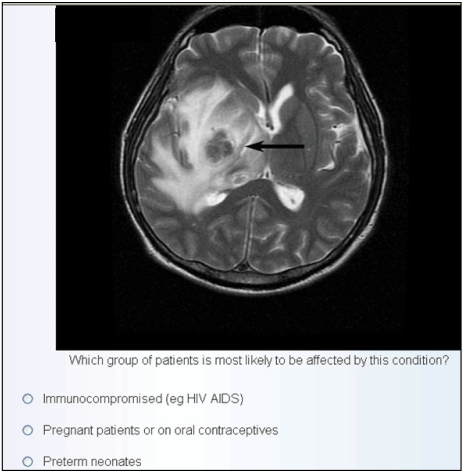
Example of a multiple-choice question based on radiological image from electronic teaching files.

Although electronic teaching files may be useful for self-directed, on-demand learning in an institutional intranet, further development is required in order to turn them into a validated (i.e. assessed) distance learning programme for CPD on the World Wide Web. Examples of validated CPD programmes would include self-assessment modules or SAMs for the American Board of Radiology [6], and assessments by CME providers who will issue pass/fail points. Teaching files form an excellent backbone for any radiology CPD programme when several cases of a particular condition or similar conditions are clustered together to form the nucleus of a learning module, for example focal lung nodules caused by tuberculosis, bronchial carcinoma, hydatid cyst. Furthermore, images in MIRC teaching files can be used for validated test questions in a distance learning CPD programme in combination with an electronic learning management system (Figure 4).

**Figure 5:**
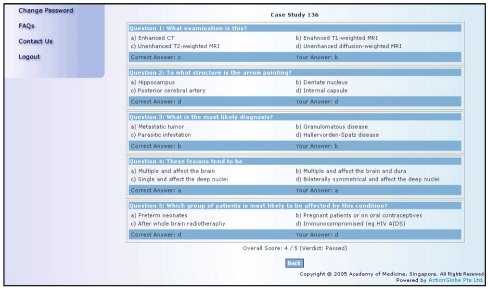
Distance learning program validated self-assessment. Display of answers to multiple choice questions is immediate and, combined with a learning management system, can support online CME.

Such learning management systems typically comprise a database of candidates with appropriate security features (such as password restricted access), and the ability to mark the candidates’ tests and decide on a score. In Singapore, efforts are being made to establish a system for radiology teaching files to participate in the CME Category 3B verifiable self-assessment distance learning programme [28]. Picture-based questions can be asked and the answers immediately assessed by the CME learning management system (Figure 5). The main advantage of using electronic teaching files created on systems such as MIRIP would be lack of copyright issues. One of the limiting factors of distance learning programmes has been the copyrighted peer-reviewed or validated content. The copyright of the teaching file in terms of images and text, however, will belong to the creator (or the institute), and such “home-grown” material would be easier to work with than copyrighted articles or image sets, which might be prohibitively expensive. An additional benefit of producing our own teaching files would be increasing expertise and improved standards of education and professional pride in the creation of high-quality intellectual content.

## MULTIMEDIA: BEYOND STATIC TEACHING FILES

Although electronic teaching files may be desirable as a first step in CPD, they are not entirely adequate for teaching all the skills required of radiological practice. For instance, merely teaching the final diagnosis limited to a static picture may not take into account the process of detection, deduction and further management inherent in all diagnostic radiology interactions. Failure to arrive at the correct multiple choice answer of the correct diagnosis may be the result of breakdown anywhere in the chain of reasoning that is taken for granted by radiologists. These include failure to detect the abnormality, to think through the different possibilities in order to rank the appropriate likely cause, or to incorporate additional non-imaging clinical information (for example, the differential diagnosis of similar-appearing nodules would be ranked differently depending on whether the patient was a sheep farmer with a fever or a smoker who works the asbestos industry).

To overcome the electronic limitations of teaching with static pictures, multimedia teaching files have been developed, representing the first step in dynamic and interactive electronic radiology teaching. Multimedia files can be used to demonstrate and explain not just the features of a particular diagnosis but also the step-wise process of arriving at such a diagnosis, making use of audio as well as moving graphics on a radiological image [29]. Furthermore, this can be applied on the World Wide Web as a discussion forum and may even be potentially useful as a form of viva voce practice for candidates preparing for an examination.

## CHALLENGES FACING RADIOLOGICAL CPD

As in all worthwhile endeavours, the usefulness and success of radiology CPD depends on the willing contribution of participants. In this case, the task would be to ensure a continuous supply of expert content, using the tools that have been developed. Despite the availability of a ready source (in PACS) and destination (in WWW and MIRIP) of images, it still requires human effort and intelligence to bring order to the information, not a trivial task in today's world of radiology staff shortage and time pressure. In many countries (some with existing shortages of radiologists) there is barely enough manpower and time to cover the ever-increasing service demands, let alone participate in CPD. To encourage healthy contribution of material, there should be CPD credit, not just for passing the assessment tests, but also for the content providers, peer reviewers and programme directors. The reality is that CPD requires participation from the whole community of practising radiologists (particularly in small communities such as in Asia), and should not be limited to those radiologists involved in teaching in University centres. The challenge before us is how to promote 'buy-in' from radiologists of today to perpetuate the admirable ethos of 'see one, do one, teach one' for the radiologists of tomorrow.

In summary, CPD is becoming an integral part of medical practice, and the radiology community faces challenges in manpower and time. With appropriate tools and mechanisms exploiting digital images, PACS and the World Wide Web, radiologists will be equal to the task of using CPD to enhance the quality of medical care.
